# Whole Blood Cytokine Response to Local Traffic-Related Particulate Matter in Peruvian Children With and Without Asthma

**DOI:** 10.3389/fphar.2017.00157

**Published:** 2017-03-30

**Authors:** Jesse P. Negherbon, Karina Romero, D’Ann L. Williams, Rafael E. Guerrero-Preston, Thomas Hartung, Alan L. Scott, Patrick N. Breysse, William Checkley, Nadia N. Hansel

**Affiliations:** ^1^Department of Environmental Health Sciences, Bloomberg School of Public Health, The Johns Hopkins University, BaltimoreMD, USA; ^2^Asociación Benéfica PrismaLima, Perú; ^3^Head and Neck Cancer Research Division, Department of Otolaryngology, School of Medicine, The Johns Hopkins University, BaltimoreMD, USA; ^4^Center for Alternatives to Animal Testing, Bloomberg School of Public Health, The Johns Hopkins University, BaltimoreMD, USA; ^5^W. Harry Feinstone Department of Molecular Microbiology and Immunology, Bloomberg School of Public Health, The Johns Hopkins University, BaltimoreMD, USA; ^6^Division of Pulmonary and Critical Care, School of Medicine, The Johns Hopkins University, BaltimoreMD, USA

**Keywords:** asthma, particulate matter, whole blood assay, inflammation, cytokines

## Abstract

This study sought to investigate if acute phase immune responses of whole blood from Peruvian children with controlled and uncontrolled asthma differed from children without asthma, following exposure to traffic-related particulate matter (TRPM). TRPM, including particulate matter from diesel combustion, has been shown to stimulate acute airway inflammation in individuals with and without asthma. For this study, a whole blood assay (WBA) was used to test peripheral whole blood samples from 27 children with asthma, and 12 without asthma. Participant blood samples were stimulated, *ex vivo*, for 24-h with an aqueous extract of TRPM that was collected near study area highways in Lima, Peru. All participant blood samples were tested against the same TRPM extract, in addition to purified bacterial endotoxin and pyrogen-free water, which served as positive and negative WBA controls, respectively. The innate and adaptive cytokine responses were evaluated in cell-free supernatants of the whole blood incubations. Comparatively similar levels were recorded for nine out of the 10 cytokines measured [e.g., – Interleukin (IL)-1β, IL-6, IL-10], regardless of study participant asthma status. However, IL-8 levels in TRPM-stimulated blood from children with uncontrolled asthma were diminished, compared to subjects without asthma (633 pg/ml vs. 1,023 pg/ml, respectively; *p* < 0.01); IL-8 responses for subjects with controlled asthma were also reduced, but to a lesser degree (799 pg/ml vs. 1,023 pg/ml, respectively; *p* = 0.10). These relationships were present before, and after, adjusting for age, sex, obesity/overweight status, C-reactive protein levels, and residential proximity to the study area’s major roadway. For tests conducted with endotoxin, there were no discernible differences in cytokine response between groups, for all cytokines measured. The WBA testing conducted for this study highlighted the capacity of the TRPM extract to potently elicit the release of IL-8 from the human whole blood system. Although the small sample size of the study limits the capacity to draw definitive conclusions, the IL-8 responses suggest that that asthma control may be associated with the regulation of a key mediator in neutrophil chemotaxis, at a systemic level, following exposure to PM derived from traffic-related sources.

## Introduction

Asthma is a respiratory disease characterized by recurrent episodes of airway inflammation, bronchial hyperreactivity, increased mucus production, and airflow obstruction (Hedlin et al., 2012; Ji et al., 2016). These symptoms may vary broadly in severity (Ray et al., 2016). Although asthma is classically associated with atopic phenotypes, and sensitization to aeroallergens, it has been estimated that only one-half of all cases are mediated by allergic, eosinophilic responses (Pearce et al., 1999; Douwes et al., 2011; Brooks et al., 2016). Consequently, there is interest in evaluating the potential of non-allergenic constituents from environmental particulate matter (PM) to trigger a worsening of asthma control (Guarnieri and Balmes, 2014; Ji et al., 2016). Respiratory exposures to PM containing such agents [e.g., – transition metals, poly-aromatic hydrocarbons (PAHs), ultrafine and nanoparticles, endotoxin], may stimulate conserved, acute-phase inflammatory activity that results in asthmatic symptoms via the production of reactive oxygen species (ROS), cytokine release, and immune cell recruitment (Williams et al., 2005; Al-Daghri et al., 2013; DeKruyff et al., 2014; Li et al., 2016).

Traffic-related PM (TRPM), including PM from diesel combustion sources, has been shown to stimulate acute airway inflammation in individuals with and without asthma (McCreanor et al., 2007; Zhang et al., 2009; Delfino et al., 2013; Alexis and Carlsten, 2014; Ji et al., 2016). TRPM exposure is regarded as an important risk factor for the development of childhood asthma, with supporting epidemiological evidence for increased prevalence of asthma morbidity amongst children who live near major highways (Venn et al., 2001; McConnell et al., 2006; Escamilla-Nuñez et al., 2008). Near roadways and urban areas, particles with aerodynamic equivalent diameters ≤ 2.5 μm (PM_2.5_) are primarily derived from combustion sources and have been associated with elevated risks of asthma and respiratory inflammation (Lee et al., 2006; Perez et al., 2012; Evans et al., 2014; Bowatte et al., 2015). Two other common traffic pollutants, ozone and nitrogen dioxide (NO_2_), have similarly been reported in mechanistic studies to stimulate acute inflammatory immune activity through ROS and oxidative stress (Bayram et al., 2001; Mirowsky et al., 2013; Wu et al., 2013). Like PM_2.5_, the asthma risks of ozone and NO_2_ are support by a variety of epidemiologic studies, which have been conducted in populations across the World (Lee et al., 2006; Alexis and Carlsten, 2014; Nishimura et al., 2016; Tétreault et al., 2016).

Ambient concentrations of PM_2.5_ and black carbon (BC) (a marker of combustion) have been found to persist within 75 – 100 meters (m) of highway traffic, and decrease to background levels after distances of 150 – 300 m (Hitchins et al., 2000; Zhu et al., 2002; Kozawa et al., 2012). The capacity for these particles to transport over long distances in the environment and deposit in the sensitive alveolar regions of the respiratory system has further fueled interest in their pro-inflammatory potential (Pope and Dockery, 2006). PM_2.5_ may also contain a high fraction of ultrafine and nano-scale particles, which are considered to be capable of translocating to the blood stream via respiratory exposure (Nemmar et al., 2004).

However, the task of evaluating the immunomodulatory characteristics of TRPM exposures is complicated by the inherently heterogeneous composition of anthropogenic, biologic, and crustal elements that may comprise the TRPM matrix (Kenyon and Liu, 2011; Esposito et al., 2014b). TRPM may carry a range of possible immunostimulatory, combustion-derived components (e.g., – PAHs and transition metals), in addition to bioaerosol constituents (Lighty et al., 2000; Sioutas et al., 2005). Both PAHs and transition metals are hallmarks of TRPM (Jeng, 2010; Pardo et al., 2015; Shuster-Meiseles et al., 2016). Currently, the immunologic response to TRPM from any single environment remains to be fully elucidated, which has limited the research community’s capacity to determine if children with asthma carry enhanced susceptibilities to TRPM-induced inflammation, as compared to children without asthma. Emerging *in vitro* methods, such as the human whole blood assay (WBA), offer opportunities to holistically assess the immunostimulatory activity of complex exposures (e.g., – TRPM) within integrated host-specific biologic systems.

In Peru, the prevalence of childhood asthma is among the highest of all Latin American countries (Mallol et al., 2000). Lima, Peru’s capitol and economic center, has developed into an expansive population center, with estimated 8.5 million people, where pervasive exposures to traffic pollutants occur throughout the city center and peripheral communities. Here, TRPM exposures may serve as a significant environmental mediator in the observed asthma morbidity. The present study was conducted in Pampas de San Juan de Miraflores (Pampas), one of the many densely populated peri-urban neighborhoods that surround Lima. Previous epidemiologic investigations conducted in Pampas by Baumann et al. (2011) indicated that children living within 100 m of a major community roadway, where mobile source emissions were most dense, carried twice the odds of reporting current asthma symptoms, compared to children living in homes distant to the road (384 m or more). Environmental monitoring was conducted in Pampas by Underhill et al. (2015), who then reported that the levels of PM_2.5_, black carbon, and NO_2_ within the indoor air of neighborhood households were often equivalent to levels detected outside of the home, in the ambient air, suggesting these traffic-related pollutants easily permeated the home environment.

For this study, we utilized a human WBA to evaluate the potential of ambient PM from this neighborhood to stimulate conserved inflammatory immune responses, and tested peripheral whole blood from local children with and without asthma. The WBA served as a unique field-applicable test with which to test individual immune responses to both standard inflammatory agents (e.g., – purified endotoxin) and complex environmental matrices (TRPM) from the local study area environment. The cytokine responses generated in the WBA were contrasted among groups of subjects, with and without asthma, in order to determine if asthma status (e.g., – no asthma, controlled asthma, uncontrolled asthma) was associated with an altered acute phase immune activity. The human whole blood system serves as a suitable reaction medium for this work as it carries an array of immunologic defense mechanisms (e.g., – innate pattern recognition receptors, complement) that recognizes a wide array of biologically relevant stimuli (Fennrich et al., 1999; Kindinger et al., 2005; Hasiwa et al., 2013; Klümper et al., 2015). Moreover, the WBA maintains the physiologic composition of the donor whole blood system (e.g., – leukocyte subpopulations, antibodies, soluble factors), and allows immune cells to respond to stimuli in their evolved cellular and molecular context (Hartung and Wendel, 1995; Schins et al., 1996).

Pro-inflammatory stimulation of whole blood results in a complex immunologic cascade that includes the rapid release of acute phase mediators (e.g., – TNFα, IL-1β, IL-6) from blood monocytes, increased cellular phagocytic activity, and the production of ROS (Mogensen, 2009; Takeuchi and Akira, 2010). The *ex vivo* incubation of human whole blood with environmental PM is an approach that is similar to other studies, which have employed monocytic cell lines and isolated peripheral blood mononuclear cells (PMBCs) to assess the cytokine inducing potential of air pollutants (Monn and Becker, 1999; Alfaro-Moreno et al., 2002; Osornio-Vargas et al., 2003). While this WBA method was originally developed to test for the presence of pyrogenic (i.e., – fever inducing) contamination in pharmaceutical and injectable drugs, it has since been adapted for the monitoring of immunomodulatory treatments (Hartung et al., 1995), the characterization of the immune status of patient groups (Diterich et al., 2001), and for environmental air quality analysis, as a novel means toward assessing the inflammatory cytokine-inducing potential of complex PM samples (Kindinger et al., 2005; Liebers et al., 2009; Bernasconi et al., 2010).

More recently, Klümper et al. (2015), utilized a WBA approach to aid in evaluating if asthma status modified the effects of air pollution exposure on whole blood cytokine response to a standard PM sample. However, the PM used in the Klümper et al. (2015) study was a standard (EHC-93) collected from Ottawa, Canada, in 1993, while the air quality measurements and study subjects were located in Wesel, Germany. Thus, the PM was not native to the subject’s local environment.

For the present study, PM_2.5_ was sampled from ambient air in the Pampas study area, at locations adjacent to the roadways that cut through the neighborhood from which our subjects were recruited. We collected filter samples of this PM and developed a single aqueous extract of stimuli for WBA tests, which were conducted using blood donations from children with and without asthma. Acute phase cytokine release was measured in peripheral whole blood, following 24-h of *ex vivo* exposure to TRPM extract, under controlled conditions. Post-incubated blood was assessed for a panel of cytokines that reflected innate and adaptive responses.

Our primary hypothesis was that levels of TRPM-stimulated acute phase inflammatory cytokines, associated with the innate immune response, would be enhanced in whole blood from children with either controlled or uncontrolled asthma, as compared to blood from children without asthma.

## Materials and Methods

### Ethics Statement

The Institutional Review Boards of the Johns Hopkins University (JHU) School of Medicine (Baltimore, MD, USA) (Study # CIR00007480) and A.B. PRISMA (Lima, Peru) approved this study. Written informed consent for participation was obtained from the parents or guardians of each participant and assent was obtained from children.

### Study Site and Population

**FIGURE 1:**
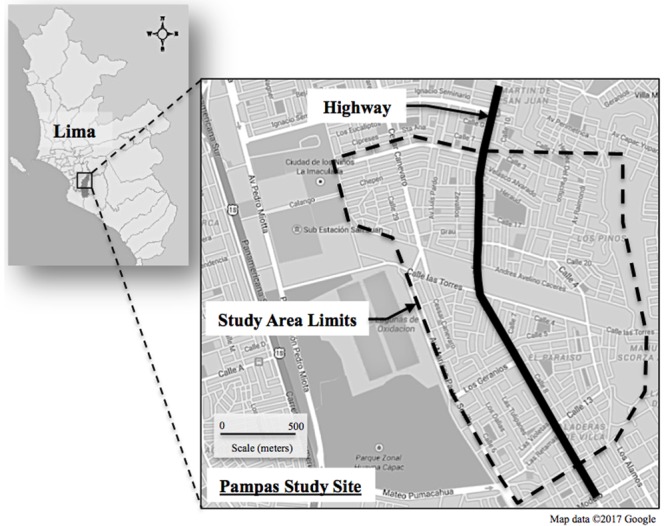
**Map of Pampas de San Juan de Miraflores location in Lima, and study area limits in relation to the highway**.

Pampas de San Juan de Miraflores is a peri-urban Peruvian neighborhood, located 25 km south of Lima’s city center (see **Figure 1**). The Pampas community encompassed low-to-middle income households, with a dense population of approximately 60,000 people (Robinson et al., 2012). Participants were recruited from a longitudinal cohort study [Genetic susceptibility to Asthma and Air Pollution Study in Peru (GASP)]. Overall, 45 children (ages 11–19) were enrolled in our study between March and June of 2014 (30 with persistent asthma and 15 controls). Enrollment was limited to one child per household and the general exclusion criteria for participants in this study included: current pregnancy, bacterial or viral infection in the past week, diagnosis of a chronic respiratory disease other than asthma (e.g., – tuberculosis, cystic fibrosis). Subjects were recruited from homes that were located either within 75 m of the major four-lane roadway in the study area, or at distances of 150 m away or greater, with the intent to enroll equal numbers of subjects living near-to and far from the roadway. Thus, the study participants do not represent a random sample from the larger GASP cohort.

Subjects with persistent asthma, and healthy controls without asthma, were confirmed based on responses recorded from a validated Asthma Screening Questionnaire (ASQ), as well as baseline spirometry tests (FEV_1_/FVC) conducted during the GASP study (collected between August of 2011 and March of 2012). The ASQ contained asthma severity questions that were derived from the International Study on Asthma and Allergies in Childhood (ISAAC) (Ellwood et al., 2005), and guidance from National Asthma Education and Prevention Program’s Expert Panel Report 3 (National Asthma Education and Prevention Program, 2007). Participants with a score < = 19 on the Asthma Control Test (ACT) were categorized as having “uncontrolled” asthma, while subjects with an ACT score ≥ 20 and <25 were considered to have “controlled” asthma. Children without asthma, or other respiratory disease, were selected as controls for the study. Questionnaire data was collected on personal tobacco cigarette use at time of enrollment in the GASP study and medication (oral and inhaled corticosteroid) use was collected in the 2 weeks prior to blood collection.

Atopy status was determined with an ImmunoCAP Phadiatop test (IummunoCAP 250-ThermoFisher Scientific, Inc.), based on three panels of allergens (animal epidermal proteins, house dust, and a mold and yeast mix). A positive result (IgE > 0.1 kUa/L) to any of the allergen panels was considered atopic. Height and weight were measured and body mass index (BMI) was calculated in kg/m^2^. Our analysis also categorized children as overweight/obese vs. normal weight, based on BMI thresholds developed by Cole et al. (2000).

### Collection and Preparation of TRPM Stimuli

Prior to the enrollment of subjects, TRPM was sampled from ambient air at five different locations within the Pampas study area, within a two-kilometer radius of each other and participant homes. PM_2.5_ samples were collected onto 37 mm polytetrafluoroethylene (PTFE) filters (Catalog #66159, Pall Corporation, Port Washington, NY, USA) using SKC PEM PM_2.5_ impactors (Catalog #761-203A, SKC Inc, Eighty-Four, PA, USA). Samplers were calibrated to run at a flow rate of 4.0 l per minute, and operated continuously for a 7-day period of time. Flow rates were checked at the beginning and end of the sampling period. Gravimetric analysis of air filters was performed at JHU, in Baltimore, MD, USA. All filters were conditioned for 24 h in a temperature and humidity controlled room to record pre- and post-sampling weights on a Mettler Toledo XP2U microbalance (Mettler Toledo, Columbus, OH, USA). Data pertaining to these filter samples are summarized in **Table 1**.

**Table 1:** Summary of TRPM filter samples.

Filter sample^a^	Mass of PM_2.5_ on Filter (mg)	Ambient PM_2.5_ concentration during sampling (μg/m^3^)
1	0.770	20
2	0.881	22
3	1.337	41
4	1.205	29
5	0.781	24

*^a^PM_2.5_ samples collected continuously over 7-day periods at flow rate of 4 l per minute.*

PM_2.5_ particles were eluted from PTFE filter membranes into a solution of pyrogen-free water (PFW) (Catalog #25-055, Mediatech Inc., Manassas, VA, USA), containing 0.05% Tween20 by volume (Catalog #BP337-500, Thermo Fisher Scientific Inc.). Air filter samples were aseptically transferred into 12 ml non-pyrogenic glass vials (Catalog #03-391-7D, ThermoFisher Scientific Inc.), using clean, gloved hands and forceps. Ten milliliters of the PFW/Tween20 solution was then pipetted into each vial to fully submerge filters in liquid. These vials were capped, vigorously mixed for 1 min, and then sonicated in a room temperature water bath (Model # 97043-964, VWR International, West Chester, PA, USA) for 60 min. Lastly, each vial was vortexed for 1 min and the entire volume of each of the filter eluates was combined in a single pyrogen-free 50 ml falcon. This solution was vortexed to thoroughly to mix the eluates, after which they were aliquoted into sterile 10 ml cryovials and frozen at -20°C for shipment to the field lab in Peru. The TRPM extract used for these WBA tests contained a particle mass concentration of 60 μg/ml, with an endotoxin content of 50 pg/ml. For reference, Klümper et al. (2015) used a PM extract at a concentration of 100 μg/ml and a review conducted by Mitschik et al. (2008), indicated that the most common PM extract concentrations used for cytokine analysis in *in vitro* cellular studies ranged between 50 and 100 μg/ml.

Bacterial endotoxin content of the pooled TRPM extract was quantified using a kinetic chromogenic Limulus Amebocyte Lysate (LAL) test (Pyrochrome^®^ Lot #2041204, Associates of Cape Cod Inc, E. Fallmouth, MA, USA), per the manufacturer’s instructions. LAL testing was conducted since endotoxin is a known pro-inflammatory agent and a contaminant of PM from many environments (Doreswamy and Peden, 2011; Matsui et al., 2013). The LAL lysate was reconstituted with a manufacture-supplied buffer (Glucashield^®^ Lot #1207034, Associates of Cape Cod Inc.) to block potential false-positive interference from β-glucans in the sample matrix. Since the kinetic chromogenic LAL test requires clear, particle-free samples for testing, 1.0 ml aliquots of the TRPM extract were centrifuged in sterile 1.5 ml microcentrifuge tubes at 1,000 *× g* for 15 min to pellet insoluble particles. Dilutions of the TRPM extract were assayed in duplicate in endotoxin-free 96-well tissue culture plates (Falcon^TM^, Corning Life Sciences, Corning, NY, USA). The LAL testing included dilutions of control standard endotoxin from *Escherichia coli* O113:H10 (concentrations of 5,000, 500, 50, 5, and 0.5 pg/ml, Lot #249021, Associates of Cape Cod Inc.), as well as media blanks and spiked samples. The limit of detection for the LAL test was 15 pg/ml.

### Whole Blood Collection and Incubation

Each participant provided a single blood donation for this study. Subjects arrived at a field lab in Pampas on Sundays, between the hours of 6 am and 10 am, where blood draws were completed within a regular schedule to control for the influence of circadian rhythms on whole blood cytokine release (Hermann et al., 2006). A certified phlebotomist collected 4.0 ml of blood into sterile blood collection tubes, which contained 75 units of sodium heparin (Catalog #367871, Becton Dickinson, Franklin Lakes, NJ, USA). Sterile 2.0 ml cryovials were pre-prepared on the mornings of each blood draw with 1.2 ml of sterile RPMI 1640 + L-Glutamine/HEPES, 300 μl of stimulus (e.g., – TRPM extract), and 300 μl of heparinized whole blood. Diluted peripheral whole blood from each study participant was challenged with the TRPM extract, as well as a positive control (Purified *E. coli* endotoxin, concentration 100 ng/ml, Lot #249020, Associates of Cape Cod Inc.), and a negative control (pyrogen-free water).

Whole blood incubations were prepared and started within 1 h of each respective participant’s blood draw. Each vial of diluted whole blood and stimulus was gently mixed by slowly inverting the vial threes times. These vials were then placed upright in a thermoblock incubator (Model # 12621-108, VWR International, West Chester, PA, USA), set to operate at 37°C. After 24 h, the blood samples were removed from the incubator, re-mixed, and then aliquoted to 1.5 ml microcentrifuge tubes. The incubated blood was immediately frozen at -20°C and transferred on the same day to a lab at the University of Cayetano (Lima, Peru) for storage at -70°C.

Aliquots of heparinized plasma were also retained following each blood draw and were frozen at -20°C for C-reactive protein (CRP) analysis. Complete blood cell (CBC) counts, with a white blood cell (WBC) differential were also completed for each participant (Medlab, Lima, Peru). All blood and plasma samples were then shipped on dry ice to labs at JHU and stored at -80°C.

### Cytokine and C-Reactive Protein Analysis

Cytokine determination was conducted using the cell-free supernatants of the whole blood incubations. Tests were completed with a high-sensitivity electrochemiluminescent multiplex cytokine assay and a 10-plex cytokine panel (V-PLEX Proinflammatory Panel 1 (human) Kit, Catalog # K15049D-1, Meso Scale Discovery, Rockville, MD, USA). This commercially available kit measured cytokines from both the innate and adaptive response, and was specific for Interleukin (IL)-1β, IL-6, Tumor Necrosis Factor alpha (TNFα), IL-8, and IL-10, as well as IL-2, IL-4, IL-13, IL-12p70, and Interferon Gamma (IFNγ). Prior to analysis, whole blood incubation samples were thawed at room temperature and then centrifuged at 10,000 *× g* for 15 min to sufficiently pellet cellular debris and particles. The lower limits of detection (LOD) for the assay were: IL-1β: 0.04 pg/ml, IL-6: 0.06 pg/ml, TNFα: 0.04 pg/ml, IL-8: 0.04 pg/ml, IL-10: 0.03 pg/ml, IL-2: 0.06 pg/ml, IL-4: 0.02 pg/ml, IL-13: 0.24 pg/ml, IL-12p70: 0.11 pg/ml, IFNγ: 0.20 pg/ml. Measurements below the LOD were assigned a value of ½ the respective detection limit.

C-reactive protein levels were quantified from plasma samples with a commercial ELISA assay (Catalog # 30-9710s, ALPCO, Salem, NH, USA), per the manufacturer’s protocol. CRP is a biomarker of systemic inflammation and may modulate whole blood cytokine response through complement activation and binding of Fc gamma receptors (Du Clos, 2013). The sensitivity of the CRP assay was 0.124 ng/ml. All cytokine and CRP analysis was completed in the Bayview Clinical Research Unit Core Laboratory (Baltimore, MD, USA).

### Statistical Analysis

Summary statistics, with measures of central tendency, were computed for each subject group, with asthma subjects dichotomized for “controlled” and “uncontrolled” asthma, based on their respective ACT scores. Asthma Group A contained subjects with controlled asthma and Group B contained subjects with uncontrolled asthma. Between-group differences were assessed with Chi-squared tests for categorical data. Continuous variables were evaluated with non-parametric Mann–Whitney *U*-tests and Kruskal–Wallis tests, with Dunn’s multiple comparisons (when more than two groups were evaluated). Multiple linear regression methods were also used to compare WBA cytokine responses between participant groups, with adjustment for age, sex, overweight/obesity status, residential proximity to roadways, and plasma CRP. Cytokine responses were natural-log transformed for use as a continuous outcome in the regression analyses. Model assumptions were assessed via residual plots and post-regression diagnostics. For all tests, the two-sided *p*-value threshold for statistical significance was set at 0.05. Statistical analysis and the creation of figures was conducted using STATA 11.2 (STATA Corp. College Station, TX, USA) and GraphPad Prism version 5 (GraphPad Software, La Jola, CA, USA).

## Results

### Study Population Characteristics

Forty-five children from Pampas were enrolled in this study. Whole blood incubations for six of the 45 study participants were discarded due to improper adjustment of temperature settings on the thermoblock incubator. Thus, WBA data was available for 27 subjects with asthma and 12 controls. Summary statistics for these participants are presented in **Table 2**. Those with asthma were more likely to be male, although there was no difference in age or BMI. Group A subjects had higher cell counts than healthy controls for total WBC, eosinophils, and monocytes. Group B subjects only differed from the control group by their eosinophil count. No participants reported the use of long-term controller medications, such as inhaled corticosteroids. One participant with uncontrolled asthma reported use of oral corticosteroids in the previous 2 weeks and one participant reported active cigarette smoking upon their enrollment in the GASP cohort.

**Table 2:** Summary of participant characteristics.

Variable^b^	Asthma^a^		Control group (N = 12)^a^	*p*-value
	Group A (*N* = 17)	Group B (*N* = 10)		
Age (years)	14 (11 – 17)	14 (12 – 19)	14 (11 – 17)	0.44ˆd
Sex (M/F)	8/2	14/3	4/8	0.01ˆc
Socioeconomic Status	0.6 (-4.4 – 1.9)	–0.9 (-4.3 – 1.6)	–1.2 (-3.3 – 1.7)	0.54ˆd
Distance to road (Near/Far)	7/10	7/3	7/5	–
BMI (kg/m^2^)	22.6 ± 5.4	24.1 ± 4.8	23.0 ± 4.4	0.58ˆd
CRP (μg/ml)	0.7 (0.1 – 2.1)	0.7 (0.2 – 3.8)	0.9 (0.1 – 2.6)	0.96ˆd
Atopy, (%)	88%	73%	67%	0.60ˆd
Cigarette smoking (N)	0	0	1	–
Oral steroid use (N)	1	0	0	–
FEV_1_/FVC (%)				
*Pre-Bronchodilator*	85.3 ± 8.0	83.5 ± 9.9^∗^	90.1 ± 4.0	0.08ˆd
*Post-Bronchodilator*	87.2 ± 6.4^∗∗^	88.5 ± 7.3^∗^	93.1 ± 4.2	0.04ˆd
FEV_1_ (% predicted)	102.9 ± 18.2^∗^	102.5 ± 19.8	110.7 ± 20.0	0.50ˆd
WBC count, (10^3^ cells/μl)	8.6 (4.8 – 9.9)^∗^	6.7 (3.8 – 11.1)	6.2 (5.2 – 8.9)	0.13ˆd
Lymphocytes	2.9 (2.0 – 4.1)	2.5 (1.5 – 4.0)	2.6 (1.9 – 3.6)	0.26ˆd
Eosinophils	0.5 (0.1 – 1.2)^∗∗^	0.6 (0.11 – 1.0)^∗∗^	0.3 (0.1 – 0.5)	0.01ˆd
Monocytes	0.6 (0.3 – 0.8)^∗^	0.4 (0.2 – 0.7)	0.4 (0.3 – 0.5)	0.04ˆd
Neutrophils	3.9 (2.0 – 7.4)	3.6 (1.8 – 7.4)	3.3 (1.8 – 4.8)	0.37ˆd
Basophils	0.0 (0.0 – 0.1)	0.0 (0.0 – 0.1)	0.0 (0.0 – 0.1)	0.76ˆd

*^a^Group A, controlled asthmatics; Group B, uncontrolled asthmatics, Control group, non-asthmatics. ^b^Data are represented at Mean ± SD, or median (range). ^c^Chi-square test. ^d^Kruskal–Wallis, post test: comparison of each Asthma group to non-asthmatic group, using Dunn’s multiple comparison (^∗^*p*-value < 0.05, ^∗∗^*p*-value < 0.01). ^e^Atopy data not available for four asthmatic participants.*

### WBA Cytokine Release

**FIGURE 2:**
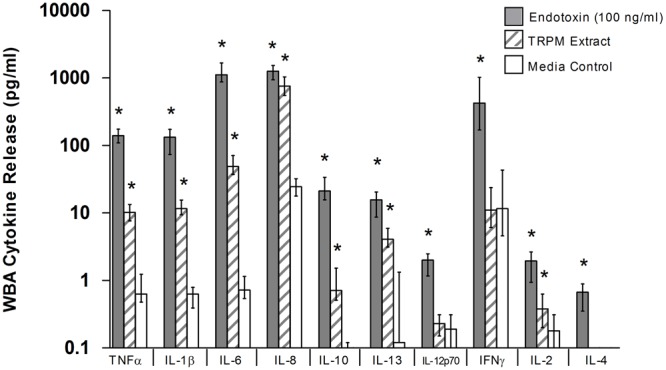
**Whole blood assay (WBA) cytokine response to 24-h stimulation with endotoxin, TRPM extract, and media control (pyrogen-free water).** TRPM-stimulated whole blood released inflammatory and anti-inflammatory cytokines at levels above the media control, but below that of the endotoxin control. Data represent median (IQR) response for all participants (*N* = 39); ^∗^*p*-value < 0.05 versus media control, Kruskal–Wallis with Dunn’s post-test for multiple comparisons (media control = reference category).

**Figure 2** summarizes WBA cytokine responses for all participants (*N* = 39), and represents the absolute levels detected in the cell-free supernatants of incubations. The TRPM-stimulated release of acute phase inflammatory cytokines was measured at levels significantly above the pyrogen-free media control for all cytokines except IL-4, IFNγ and IL-12p70, which were therefore excluded from further analysis. Overall, the cytokine-stimulating potential of the TRPM extract was less potent than that of the endotoxin control.

**Table 3** summarizes TRPM, PFW, and endotoxin-stimulated cytokine responses for healthy controls and Asthma Groups A and B (those subjects with controlled and uncontrolled asthma, respectively). For most cytokines, Group B subjects were associated with the lowest cytokine responses for WBA tests conducted with TRPM extract, while response estimates for Group A subjects were measured at levels between those of Group B and the healthy controls. In particular, IL-8 response levels in TRPM-stimulated blood from Group B subjects were diminished, in comparison to the control group (633 pg/ml vs. 1,023 pg/ml, respectively; Mann–Whitney *U*-Test, *p* < 0.01). IL-8 estimates for Group A subjects were also reduced from controls, but to a lesser degree (799 pg/ml vs. 1,023 pg/ml, respectively; *p* = 0.10). For WBA tests conducted with endotoxin, no clear trend in cytokine response was evident between the asthma and control groups.

**Table 3:** Whole blood assay cytokine response.

	Stimulus: TRPM Extract				Positive Control: Endotoxin (100 ng/ml)				Negative Control: Pyrogen-free Water			
		Asthma^a^				Asthma^a^				Asthma		*p*- value
Cytokine (pg/ml)^b^	Control group (*N* = 12)	Group A (*N* = 10)	Group B (*N* = 17)	*p-*value	Control group (*N* = 12)	Group A (*N* = 10)	Group B (*N* = 17)	*p-*value	Control group (*N* = 12)	Group A (*N* = 10)	Group B (*N* = 17)	
TNFα	11.70	10.83	8.56	0.21	159.83	132.47	129.09	0.56	0.90	0.55	0.62	0.15
	(12.78)	(4.79)	(7.61)*		(56.52)	(70.00)	(46.05)		(1.57)	(0.72)	(0.40)	
IL-1β	11.89	12.48	9.78	0.86	133.07	112.65	132.93	0.99	0.58	0.59	0.65	0.92
	(9.65)	(4.12)	(6.15)		(111.91)	(96.81)	(92.89)		(0.58)	(0.15)	(0.28)	
IL-6	65.62	49.95	46.09	0.38	1,238.6	1,468.7	1,071.1	0.56	0.78	0.68	0.74	0.74
	(57.28)	(29.82)	(14.72)		(860.2)	(837.2)	(273.9)		(0.71)	(0.32)	(0.49)	
IL-8	1023.4	798.6	633.3	0.02	1,299.7	1,125.9	1,273.5	0.66	29.96	23.82	23.34	0.12
	(975.1)	(351.8)	(311.9)*		(718.3)	(675.4)	(549.9)		(25.84)	(10.72)	(13.12)*	
IL-10	0.86	0.82	0.59	0.65	21.97	21.05	20.59	0.66	0.12	0.04	0.06	0.12
	(0.77)	(1.24)	(0.67)		(23.05)	(16.15)	(17.40)		(0.08)	(0.08)*	(0.07)	
IL-13	5.57	3.59	3.88	0.22	19.84	10.12	15.69	0.20	1.48	<LOD	<LOD	–
	(3.88)	(2.12)*	(3.22)		(16.59)	(11.53)*	(7.99)		(1.45)			
IL-2	0.37	0.30	0.46	0.98	1.83	1.98	2.15	0.66	0.26	0.15	0.13	0.06
	(0.40)	(0.41)	(0.50)		(1.34)	(4.21)	(1.91)		(0.26)	(0.19)*	(0.24)*	

*^a^Group A, controlled asthma; Group B, uncontrolled asthma; Control Group, No asthma. ^b^Data represent median and interquartile range (IQR). ^c^Cytokine levels measured in supernatant of whole blood incubations. ^∗^*p*-value < 0.05; Kruskal–Wallis test, with Dunn’s multiple comparison post test (control group = reference).*

**Table 4** summarizes Spearman rank-order correlation coefficients between cytokine responses of WBA tests conducted with endotoxin (the positive control) and TRPM. While modest evidence of correlation exists for cytokines such at TNF-α, IL-1β, and IL-6, there is a notable lack of correlation evident for the IL-8 responses to endotoxin and TRPM, whether the study subjects were considered as a single group, or as sub-groups based on asthma control status. Overall, this relationship, or lack thereof, suggests that a participant’s IL-8 response to endotoxin was not predictive of the IL-8 response to the TRPM extract.

**Table 4:** Spearman’s correlation between TRPM and endotoxin WBA responses.

Cytokine (pg/ml)^3^	Spearman’s Rho for all subjects (*N* = 39)	Spearman’s Rho (*p*-value)		
		Control group (*N* = 12)	Asthma Group A (*N* = 10)	Asthma Group B (*N* = 17)
TNFα	0.39 (0.01)	0.63 (0.03)	0.39 (0.26)	0.15 (0.56)
IL-1β	0.53 (0.01)	0.57 (0.05)	0.09 (<0.01)	0.40 (0.12)
IL-6	0.39 (0.01)	0.30 (0.34)	0.54 (0.11)	0.14 (0.60)
IL-8	–0.04 (0.82)	–0.19 (0.56)	–0.13 (0.73)	0.02 (0.93)
IL-10	0.48 (<0.01)	0.83 (<0.01)	0.88 (<0.01)	0.11 (0.68)
IL-13	0.19 (0.25)	0.29 (0.35)	–0.21 (0.56)	0.11 (0.68)
IL-2	0.46 (<0.01)	0.41 (0.19)	0.67 (0.03)	0.33 (0.19)

Whole blood assay cytokine responses were also investigated using multiple linear regression analysis to evaluate relationships between asthma control status and cytokine release. We observed no difference in the release of TNFα, IL-1β, IL-6, IL-10, IL-2, or IL-13 between the control group and either Groups A or B. For IL-8, subjects from Group B, with uncontrolled asthma, had 37% (β = -0.47, e^-0.47^= 0.63, *p* = 0.01) lower responses (geometric mean) to TRPM stimulation than the control group, without asthma. Group A subjects, with controlled asthma, had an intermediate IL-8 response between control subjects and those in Group B. There was no statistically significant difference in TRPM-stimulated IL-8 response between subjects with Group A and those without asthma (β = -0.28, *p* = 0.17).

## Discussion

The present study investigated the systemic immune function of children with and without asthma through *ex vivo* stimulation of peripheral whole blood immune cells. In particular, we evaluated whether blood from individuals with uncontrolled asthma had differential acute phase inflammatory cytokine responses to stimulation with an aqueous extract of local study area TRPM, compared to blood obtained from individuals with controlled asthma or those without asthma. *Ex vivo* whole blood exposure to the TRPM extract produced increased levels of acute phase inflammatory mediators (including TNF-α, IL-1β, IL-6, IL-8) in blood samples from all study participants.

However, contrary to our *a priori* hypothesis, we found that TRPM-stimulated WBA cytokine responses were generally equivalent across subject groups, regardless of asthma control status. The most notable exception was for TRPM-stimulated levels of IL-8, where children with uncontrolled asthma were found to have the lowest levels, both before and after adjusting for age, sex, obesity/overweight status. The relationship between IL-8 responses and the asthma control category was also not influenced by plasma CRP levels, or residential proximity to the study area’s major roadway. Similarly, IL-8 response estimates for subjects with controlled asthma were less pronounced than those of the control group, although the difference was not statistically significant. For *ex vivo* WBA tests conducted with purified bacterial endotoxin, there were no discernable differences between groups, for any of the cytokines that were measured.

The WBA testing conducted for this study highlighted the capacity of the TRPM extract to potently elicit the release of IL-8 from the human whole blood system. IL-8 is an important inflammatory mediator, *in vivo*, with effects that include signaling for chemotaxis, especially for neutrophils, and the promotion of angiogenesis (Kunkel et al., 1991). Elevated levels of IL-8 in fluids from the respiratory environment are closely associated with the neutrophilic asthma phenotype, among other inflammatory diseases (Simpson et al., 2008). However, it is also noted that IL-8 polymorphisms have been associated with differential IL-8 gene expression and disease [e.g., - sepsis (Hu et al., 2016), viral bronchiolitis (Pinto et al., 2016)]. In the case of asthma, Esposito et al. (2014a) have previously reported an association between an IL-8 polymorphism and increased odds of wheezing among infants. Thus, we acknowledge that the genetics, and epigenetics, of participants in this study may have influenced the observed IL-8 responses in the WBA. Moreover, it reinforces the complexity of the challenges associated with isolating and evaluating the environmental, genetic, and epigenetic factors associated with asthma.

Previous studies have shown that ambient PM provokes the release of IL-8 from a variety of human cell types, including airway epithelial cells and those of monocytic lineage (Steerenberg et al., 1998; Veranth et al., 2006; Wu et al., 2014). *In vitro* testing has also provided evidence to suggest that ambient PM, with low or negligible levels of endotoxin, elicits the release of IL-8 from immune cells through the non-canonical activation of nuclear factor kappa beta (NFκB), primarily driven by oxidative stress (Silbajoris et al., 2011), as opposed to pattern recognition receptor engagement (e.g., – Toll-like Receptor 4) (Akira et al., 2006). The TRPM samples used to develop the extract for our study carried a low burden of endotoxin, and thus we believe that non-endotoxin constituents in this PM were responsible for the pro-inflammatory effects observed in our WBA tests. We acknowledge that our current results are unable to distinguish the specific constituents of TPRM that primarily contribute to immunogenicity. However, as mentioned above, TRPM is a complex mixture and a key strength of the WBA assay is its ability to assess integrated responses to complex exposures.

Our results align with previous findings from other research groups, who have reported attenuated IL-8 levels in sputum, nasal lavage, and bronchoalveolar wash from TRPM-exposed subjects with asthma, as compared to similarly exposed healthy controls (Stenfors et al., 2004; Barraza-Villarreal et al., 2008; Behndig et al., 2011). However, the results of our WBA testing do not provide mechanistic support for other epidemiological studies, which have observed an association between ambient traffic pollution exposure and enhanced respiratory sensitivity (e.g., – bronchoconstriction, wheeze) in asthmatic subjects (Holguin et al., 2007; Escamilla-Nuñez et al., 2008).

In the context of the WBA model, our analysis is most similar to that of Klümper et al. (2015), which is the only study (to our knowledge) that has used similar whole blood methods to assess the inflammatory effects of PM exposure in children with and without asthma. The authors of this study used a PM standard (collected from ambient air in Ottawa, ON, Canada), to stimulate peripheral whole blood collected from 6-year old children, with and without asthma, who lived in Germany. Similar to our findings, the authors observed that whole blood stimulation with urban PM induced the release of pro-inflammatory cytokines (including IL-6, IL-8, and TNF-α), and no difference in responsiveness to PM stimulation was found between children with and without asthma (Klümper et al., 2015). Interestingly, the authors reported that among asthmatics, NO_2_ exposure (estimated via a land-use regression modeled) was positively associated with increased levels of IL-6 from urban PM-stimulated whole blood, suggesting that chronic traffic pollutant exposure may enhance the innate cytokine response to PM in blood from children with asthma.

A primary limitation of the Klümper et al. (2015) study was its small sample size, which included 27 asthmatic subjects and 59 control subjects without asthma. The design and fieldwork for our study was conducted before publication of the Klümper et al. (2015) study, and we emphasize that small sample size was also the primary limitiation of our study, which may have limited our capacity to detect smaller differences in cytokine responses that may have been present between subject groups. An increased sample size, and repeat WBA testing among study subjects would strengthen the capacity to identify differences between subject groups. The cross-sectional approach used for WBA testing in this study, prevented us from evaluating within-subject variability for WBA responses over time. As a result, we could not evaluate the temporal robustness of cytokine response trends between asthma and controls groups, or the variability of TRPM pro-inflammatory potential over time.

We are also mindful that our study was conducted using a single aqueous extract of PM_2.5_ particles, derived from samples collected in the community where the children lived, over a 2-week period in 2014. In one manner, the evaluation of these children’s systemic responsiveness to TRPM derived from their local environment was a unique strength of this study and the results add to the existing literature base on traffic pollution and pediatric asthma. However, we also acknowledge that the composition of ambient PM is known to vary over time in any single environment, and thus the pro-inflammatory potential of PM may fluctuate as well (Manzano-León et al., 2013; Quintana-belmares et al., 2015). The time-integrated TRPM sampling conducted for this study may not be representative of TRPM from other times during the year, or from other environments.

The relationship between WBA cytokine release and the local airway responsiveness of asthmatics requires further study, particularly given our observation of depressed IL-8 responses to TRPM exposure in subjects with uncontrolled asthma. The measurement of exhaled breath condensate (EBC) biomarkers (e.g., – pH, cytokine, NO, matrix metalloproteases) may have provided added depth to assess and contrast relationships between WBA cytokine responses, asthma severity, peripheral blood cell counts, and markers of airway inflammation. Previous studies have associated a variety of EBC biomarkers with asthma severity and lung function (Ueno et al., 2008; Karakoc et al., 2012; Turkeli et al., 2015) While many of these EBC biomarkers remain a developing science, they may serve as a unique complement to WBA cytokine data, such as ours. It is important to determine if WBA cytokine responses to environmental constituents are predictive of airway and respiratory responsiveness to the same exposures.

We do acknowledge that immune cells in the peripheral circulation are relatively short-lived and have a rapid turnover, which may not accurately mirror the reactivity status of the airways. While our study results indicate that asthma control was associated with a differentially lower IL-8 response to TRPM exposure, additional mechanistic study is necessary (beyond WBA testing) to delineate the pathways activated by complex environmental stimuli, such as the TRPM extract used in this study. Genotyping candidate genes in participant whole blood would also allow for cytokine responses to be evaluated as a function of potential genetic polymorphisms.

The literature on cytokine changes in asthma remains a subject of debate, and serum IL-8 is regarded as a poor indicator of disease activity in acute asthma (Tang and Chen, 2000). Our findings of reduced release capacity of stimulated blood in asthmatic donors might indicate that IL-8 release has “burned out” or was counterregulated. Stenfors et al. (2004) exposed healthy and asthmatic subjects to diesel PM and reported that asthmatic subjects had eosinophilic, rather than neutrophilic inflammation, and that IL-10 levels in staining of lung epithelial cells were increased. However, we did not see significantly higher IL-10 levels in exposed blood from our subjects, probably due to the small sample size.

Depressed IL-8 responses in the whole blood system may also suggest impaired cellular phagocytosis, oxidative burst, and neutrophil chemotaxis, which could compromise the strength of innate immune defenses against microbial infection (Mayadas et al., 2014). If such effects extend beyond the whole blood system, and impact localized respiratory cell populations, they may result in compromised respiratory immune defense to infection.

## Conclusion

This study provided insight toward the capacity of TRPM to elicit the release of acute phase pro-inflammatory mediators from the whole blood systems of Peruvian children. We observed comparatively similar levels of acute inflammatory markers (e.g., – IL-1β, IL-6, TNFα) in TRPM-stimulated whole blood from children with and without asthma, which was contrary to our *a priori* study hypothesis. However, for WBA tests conducted with TRPM stimuli, we found that subjects with uncontrolled asthma were associated with lower levels of IL-8, on average, as compared to children without asthma. Although the small sample size of our study limits definitive conclusions, the IL-8 responses from our testing suggest that that asthma control may be associated with the regulation of a key mediator in neutrophil chemotaxis, at a systemic level, following exposure to PM derived from traffic-related sources. However, there remains a need to further evaluate the correlation between acute phase WBA cytokine response and measures of asthmatic and non-asthmatic airway inflammation.

## Author Contributions

NH, WC, RG-P, TH, DW, and PB contributed to the grant proposal and conceptual design for this study. KR directed subject recruitment, blood draws, data and field lab management, and the Spanish translation of study protocols. JN and KR trained field staff for the whole blood assay testing and conducted the statistical analysis of the data. JN, NH, AS, DW, TH, and PB prepared the manuscript, which was reviewed and approved by all other authors, prior to journal submission.

## Conflict of Interest Statement

It is declared that TH holds patents for the whole blood assay pyrogen test used in this study and described in the body of the manuscript. TH also holds patents for whole blood pyrogen tests completed with cryopreserved blood, which are also referenced in the manuscript. TH is also an Associate Editor for Frontiers in Pharmacology. The other authors declare that the research was conducted in the absence of any commercial or financial relationships that could be construed as a potential conflict of interest.

## References

[B1] Akira S. Uematsu S. Takeuchi O. (2006). Pathogen recognition and innate immunity. *Cell* 124 783–801. 10.1016/j.cell.2006.02.015 16497588

[B2] Al-Daghri N. M. Alokail M. S. Abd-Alrahman S. H. Draz H. M. Yakout S. M. Clerici M. (2013). Polycyclic aromatic hydrocarbon exposure and pediatric asthma in children: a case–control study. *Environ. Heal.* 12:1 10.1186/1476-069X-12-1 PMC3621697 23286340

[B3] Alexis N. E. Carlsten C. (2014). Interplay of air pollution and asthma immunopathogenesis: a focused review of diesel exhaust and ozone. *Int. Immunopharmacol.* 23 347–355. 10.1016/j.intimp.2014.08.009 25194677

[B4] Alfaro-Moreno E. Martínez L. García-Cuellar C. Bonner J. C. Murray J. C. Rosas I. (2002). Biologic effects induced in vitro by PM10 from three different zones of Mexico City. *Environ. Health Perspect.* 110 715–720. 10.1289/ehp.02110715 12117649 PMC1240918

[B5] Barraza-Villarreal A. Sunyer J. Hernandez-Cadena L. Escamilla-Nuñez M. C. Sienra-Monge J. J. Ramírez-Aguilar M. (2008). Air pollution, airway inflammation, and lung function in a cohort study of Mexico City schoolchildren. *Environ. Health Perspect.* 116 832–838. 10.1289/ehp.10926 18560490 PMC2430242

[B6] Baumann L. M. Robinson C. L. Combe J. M. Gomez A. Romero K. Gilman R. H. (2011). Effects of distance from a heavily transited avenue on asthma and atopy in a periurban shantytown in Lima, Peru. *J. Allergy Clin. Immunol.* 127 875–882. 10.1016/j.jaci.2010.11.031 21237505 PMC3227546

[B7] Bayram H. Sapsford R. J. Abdelaziz M. M. Khair O. A. (2001). Effect of ozone and nitrogen dioxide on the release of proinflammatory mediators from bronchial epithelial cells of nonatopic nonasthmatic subjects and atopic asthmatic patients in vitro. *J. Allergy Clin. Immunol.* 107 287–294. 10.1067/mai.2001.111141 11174195

[B8] Behndig A. F. Larsson N. Brown J. L. Stenfors N. Helleday R. Duggan S. T. (2011). Proinflammatory doses of diesel exhaust in healthy subjects fail to elicit equivalent or augmented airway inflammation in subjects with asthma. *Thorax* 66 12–19. 10.1136/thx.2010.140053 20837873

[B9] Bernasconi C. Rodolfi M. Picco A. M. Grisoli P. Dacarro C. Rembges D. (2010). Pyrogenic activity of air to characterize bioaerosol exposure in public buildings: a pilot study. *Lett. Appl. Microbiol.* 50 571–577. 10.1111/j.1472-765X.2010.02831.x 20337928

[B10] Bowatte G. Lodge C. Lowe A. J. Erbas B. Perret J. Abramson M. J. (2015). The influence of childhood traffic-related air pollution exposure on asthma, allergy and sensitization: a systematic review and a meta-analysis of birth cohort studies. *Allergy* 70 245–256. 10.1111/all.12561 25495759

[B11] Brooks C. R. van Dalen C. J. Zacharasiewicz A. Simpson J. L. Harper J. L. Le Gros G. (2016). Absence of airway inflammation in a large proportion of adolescents with asthma. *Respirology* 21 460–466. 10.1111/resp.12701 26693952

[B12] Cole T. J. Bellizzi M. C. Flegal K. M. Dietz W. H. (2000). Establishing a standard definition for child overweight and obesity worldwide: international survey. *BMJ* 320 1240–1243. 10.1136/bmj.320.7244.1240 10797032 PMC27365

[B13] DeKruyff R. H. Yu S. Kim H. Y. Umetsu D. T. (2014). Innate immunity in the lung regulates the development of asthma. *Immunol. Rev.* 260 235–248. 10.1111/imr.12187 24942693

[B14] Delfino R. J. Staimer N. Tjoa T. Gillen D. L. Schauer J. J. Shafer M. M. (2013). Airway inflammation and oxidative potential of air pollutant particles in a pediatric asthma panel. *J. Expo. Sci. Environ. Epidemiol.* 23 466–473. 10.1038/jes.2013.25 23673461 PMC4181605

[B15] Diterich I. Härter L. Hassler D. Wendel A. Hartung T. (2001). Modulation of cytokine release in ex vivo-stimulated blood from borreliosis patients. *Infect. Immun.* 69 687–694. 10.1128/IAI.69.2.687-694.2001 11159956 PMC97940

[B16] Doreswamy V. Peden D. B. (2011). Modulation of asthma by endotoxin. *Clin. Exp. Allergy* 41 9–19. 10.1111/j.1365-2222.2010.03628.x 20977505

[B17] Douwes J. Brooks C. van Dalen C. Pearce N. (2011). Importance of allergy in asthma: an epidemiologic perspective. *Curr. Allergy Asthma Rep.* 11 434–444. 10.1007/s11882-011-0215-6 21792637

[B18] Du Clos T. W. (2013). Pentraxins: structure, function, and role in inflammation. *ISRN Inflamm.* 2013:379040 10.1155/2013/379040 PMC3791837 24167754

[B19] Ellwood P. Asher M. I. Beasley R. Clayton T. O. Stewart A. W. (2005). The international study of asthma and allergies in childhood (ISAAC): phase three rationale and methods. *Int. J. Tuberc. Lung Dis.* 9 10–16. 15675544

[B20] National Asthma Education and Prevention Program (2007). Expert Panel Report 3 (National Asthma Education and Prevention Program): guidelines for the diagnosis and management of asthma-summary report 2007. *J. Allergy Clin. Immunol.* 120 S94–138. 10.1016/j.jaci.2007.09.043 17983880

[B21] Escamilla-Nuñez M.-C. Barraza-Villarreal A. Hernandez-Cadena L. Moreno-Macias H. Ramirez-Aguilar M. Sienra-Monge J.-J. (2008). Traffic-related air pollution and respiratory symptoms among asthmatic children, resident in Mexico City: the EVA cohort study. *Respir. Res.* 9:74 10.1186/1465-9921-9-74 PMC2613139 19014608

[B22] Esposito S. Ierardi V. Daleno C. Scala A. Terranova L. Tagliabue C. (2014a). Genetic polymorphisms and risk of recurrent wheezing in pediatric age. *BMC Pulm. Med.* 14:162 10.1186/1471-2466-14-162 PMC4210469 25326706

[B23] Esposito S. Tenconi R. Lelii M. Preti V. Nazzari E. Consolo S. (2014b). Possible molecular mechanisms linking air pollution and asthma in children. *BMC Pulm. Med.* 14:31 10.1186/1471-2466-14-31 PMC3941253 24581224

[B24] Evans K. A. Halterman J. S. Hopke P. K. Fagnano M. Rich D. Q. (2014). Increased ultrafine particles and carbon monoxide concentrations are associated with asthma exacerbation among urban children. *Environ. Res.* 129 11–19. 10.1016/j.envres.2013.12.001 24528997 PMC3947881

[B25] Fennrich S. Wendel A. Hartung T. (1999). New applications of the human whole blood pyrogen assay (PyroCheck). *ALTEX* 16 146–149. 11107322

[B26] Guarnieri M. Balmes J. R. (2014). Outdoor air pollution and asthma. *Lancet* 383 1581–1592. 10.1016/S0140-6736(14)60617-6 24792855 PMC4465283

[B27] Hartung T. Döcke W. D. Gantner F. Krieger G. Sauer A Stevens P. (1995). Effect of granulocyte colony-stimulating factor treatment on ex vivo blood cytokine response in human volunteers. *Blood* 85 2482–2489. 7537116

[B28] Hartung T. Wendel A. (1995). [Detection of Pyrogens using human whole blood]. *ALTEX* 12 70–75. 11178418

[B29] Hasiwa N. Daneshian M. Bruegger P. Fennrich S. Hochadel A. Hoffmann S. (2013). Evidence for the detection of non-endotoxin pyrogens by the whole blood monocyte activation test. *ALTEX* 30 169–208. 10.14573/altex.2013.2.169 23665806

[B30] Hedlin G. Konradsen J. Bush A. (2012). An update on paediatric asthma. *Eur. Respir. Rev.* 21 175–185. 10.1183/09059180.00003212 22941882 PMC9487327

[B31] Hermann C. von Aulock S. Dehus O. Keller M. Okigami H. Gantner F. (2006). Endogenous cortisol determines the circadian rhythm of lipopolysaccharide–but not lipoteichoic acid–inducible cytokine release. *Eur. J. Immunol.* 36 371–379. 10.1002/eji.200535470 16453387

[B32] Hitchins J. Morawska L. Wolff R. Gilbert D. (2000). Concentrations of submicrometre particles from vehicle emissions near a major road. *Atmos. Environ.* 34 51–59. 10.1016/S1352-2310(99)00304-0

[B33] Holguin F. Flores S. Ross Z. Cortez M. Molina M. Molina L. (2007). Traffic-related exposures, airway function, inflammation, and respiratory symptoms in children. *Am. J. Respir. Crit. Care Med.* 176 1236–1242. 10.1164/rccm.200611-1616OC 17641154

[B34] Hu D. Wang H. Huang X. Jiang Y. Qin Y. Xiong B. (2016). Investigation of association between IL-8 serum levels and IL8 polymorphisms in Chinese patients with sepsis. *Gene* 594 165–170. 10.1016/j.gene.2016.09.024 27642120

[B35] Jeng H. A. (2010). Chemical composition of ambient particulate matter and redox activity. *Environ. Monit. Assess.* 169 597–606. 10.1007/s10661-009-1199-8 19902370

[B36] Ji H. Biagini Myers J. M. Brandt E. B. Brokamp C. Ryan P. H. Khurana Hershey G. K. (2016). Air pollution, epigenetics, and asthma. *Allergy Asthma Clin. Immunol.* 12 51 10.1186/s13223-016-0159-4 PMC5069789 27777592

[B37] Karakoc G. B. Yukselen A. Yilmaz M. Altintas D. U. Kendirli S. G. (2012). Exhaled breath condensate MMP-9 level and its relationship wıth asthma severity and interleukin-4/10 levels in children. *Ann. Allergy. Asthma Immunol.* 108 300–304. 10.1016/j.anai.2012.02.019 22541398

[B38] Kenyon N. Liu F.-T. (2011). Pulmonary effects of diesel exhaust: neutrophilic inflammation, oxidative injury, and asthma. *Am. J. Pathol.* 179 2678–2682. 10.1016/j.ajpath.2011.08.031 22005277 PMC3263604

[B39] Kindinger I. Daneshian M. Baur H. Gabrio T. Hofmann A. Fennrich S. (2005). A new method to measure air-borne pyrogens based on human whole blood cytokine response. *J. Immunol. Methods* 298 143–153. 10.1016/j.jim.2005.01.006 15847804

[B40] Klümper C. Krämer U. Lehmann I. von Berg A. Berdel D. Herberth G. (2015). Air pollution and cytokine responsiveness in asthmatic and non-asthmatic children. *Environ. Res.* 138 381–390. 10.1016/j.envres.2015.02.034 25769127

[B41] Kozawa K. H. Winer A. M. Fruin S. A. (2012). Ultrafine particle size distributions near freeways: effects of differing wind directions on exposure. *Atmos. Environ.* 63 250–260. 10.1016/j.atmosenv.2012.09.045 PMC3886859 24415904

[B42] Kunkel S. L. Standiford T. Kasahara K. Strieter R. M. (1991). Interleukin-8 (IL-8): the major neutrophil chemotactic factor in the lung. *Exp. Lung Res.* 17 17–23. 10.3109/01902149109063278 2013270

[B43] Lee S. L. Wong W. H. S. Lau Y. L. (2006). Association between air pollution and asthma admission among children in Hong Kong. *Clin. Exp. Allergy* 36 1138–1146. 10.1111/j.1365-2222.2006.02555.x 16961713 PMC1618810

[B44] Li N. Georas S. Alexis N. Fritz P. Xia T. Williams M. A. (2016). A work group report on ultrafine particles (Awa &amp; Immunology): why ambient ultrafine and engineered nanoparticles should receive special attention for possible adverse health outcomes in human subjects. *J. Allergy Clin. Immunol.* 138 386–396. 10.1016/j.jaci.2016.02.023 27130856 PMC4976002

[B45] Liebers V. Stubel H. Düser M. Brüning T. Raulf-Heimsoth M. (2009). Standardization of whole blood assay for determination of pyrogenic activity in organic dust samples. *Int. J. Hyg. Environ. Health* 212 547–556. 10.1016/j.ijheh.2009.03.003 19395310

[B46] Lighty J. S. Veranth J. M. Sarofim A. F. (2000). Combustion aerosols: factors governing their size and composition and implications to human health. *J. Air Waste Manag. Assoc.* 50 1565–1618. 10.1080/10473289.2000.10464197 11055157

[B47] Mallol J. Solé D. Asher I. Clayton T. Stein R. Soto-Quiroz M. (2000). Prevalence of asthma symptoms in Latin America: the International Study of Asthma and Allergies in Childhood (ISAAC). *Pediatr. Pulmonol.* 30 439–444. 10.1002/1099-0496(200012)30:6<439::AID-PPUL1>3.0.CO;2-E 11109054

[B48] Manzano-León N. Quintana R. Sánchez B. Serrano J. Vega E. Vázquez-López I. (2013). Variation in the composition and in vitro proinflammatory effect of urban particulate matter from different sites. *J. Biochem. Mol. Toxicol.* 27 87–97. 10.1002/jbt.21471 23335408 PMC4355014

[B49] Matsui E. C. Hansel N. N. Aloe C. Schiltz A. M. Peng R.D. Rabinovitch N. (2013). Indoor pollutant exposures modify the effect of airborne endotoxin on asthma in urban children. *Am. J. Respir. Crit. Care Med.* 188 1210–1215. 10.1164/rccm.201305-0889OC 24066676 PMC3863732

[B50] Mayadas T. N. Cullere X. Lowell C. A. (2014). The multifaceted functions of neutrophils. *Annu. Rev. Pathol.* 9 181–218. 10.1146/annurev-pathol-020712-164023 24050624 PMC4277181

[B51] McConnell R. Berhane K. Yao L. Jerrett M. Lurmann F. Gilliland F. (2006). Traffic, susceptibility, and childhood asthma. *Environ. Health Perspect.* 114 766–772. 10.1289/ehp.8594 16675435 PMC1459934

[B52] McCreanor J. Cullinan P. Nieuwenhuijsen M. J. Stewart-Evans J. Malliarou E. Jarup L. (2007). Respiratory effects of exposure to diesel traffic in persons with asthma. *N. Engl. J. Med.* 357 2348–2358. 10.1056/NEJMoa071535 18057337

[B53] Mirowsky J. Hickey C. Horton L. Blaustein M. Galdanes K. Peltier R. E. (2013). The effect of particle size, location and season on the toxicity of urban and rural particulate matter. *Inhal. Toxicol.* 25 747–757. 10.3109/08958378.2013.846443 24255952 PMC3972067

[B54] Mitschik S. Schierl R. Nowak D. Jörres R. A. (2008). Effects of particulate matter on cytokine production in vitro: a comparative analysis of published studies. *Inhal. Toxicol.* 20 399–414. 10.1080/08958370801903784 18302048

[B55] Mogensen T. H. (2009). Pathogen recognition and inflammatory signaling in innate immune defenses. *Clin. Microbiol. Rev.* 22 240–273. 10.1128/CMR.00046-08 19366914 PMC2668232

[B56] Monn C. Becker S. (1999). Cytotoxicity and induction of proinflammatory cytokines from human monocytes exposed to fine (PM2.5) and coarse particles (PM10-2.5) in outdoor and indoor air. *Toxicol. Appl. Pharmacol.* 155 245–252. 10.1006/taap.1998.8591 10079210

[B57] Nemmar A. Hoylaerts M. F. Hoet P. H. M. Nemery B. (2004). Possible mechanisms of the cardiovascular effects of inhaled particles: systemic translocation and prothrombotic effects. *Toxicol. Lett.* 149 243–253. 10.1016/j.toxlet.2003.12.061 15093270

[B58] Nishimura K. K. Iwanaga K. Oh S. S. Pino-Yanes M. Eng C. Keswani A. (2016). Early-life ozone exposure associated with asthma without sensitization in Latino children. *J. Allergy Clin. Immunol.* 138 1703–1706.e1 10.1016/j.jaci.2016.03.058 27423496 PMC5148667

[B59] Osornio-Vargas A. R. Bonner J. C. Alfaro-Moreno E. Martínez L. García-Cuellar C. Ponce-de-León Rosales S. (2003). Proinflammatory and cytotoxic effects of Mexico City air pollution particulate matter in vitro are dependent on particle size and composition. *Environ. Health Perspect.* 111 1289–1293. 10.1289/ehp.5913 12896848 PMC1241608

[B60] Pardo M. Shafer M. M. Rudich A. Schauer J. J. Rudich Y. (2015). Single exposure to near roadway particulate matter leads to confined inflammatory and defense responses: possible role of metals. *Environ. Sci. Technol.* 49 8777–8785. 10.1021/acs.est.5b01449 26121492

[B61] Pearce N. Pekkanen J. Beasley R. (1999). How much asthma is really attributable to atopy? *Thorax* 54 268–272. 10.1136/thx.54.3.268 10325905 PMC1745435

[B62] Perez L. Lurmann F. Wilson J. Pastor M. Brandt S. J. Künzli N. (2012). Near-roadway pollution and childhood asthma: implications for developing “Win–Win” compact urban development and clean vehicle strategies. *Environ. Health Perspect.* 120 1619–1626. 10.1289/ehp.1104785 23008270 PMC3556611

[B63] Pinto L. A. De Azeredo Leitão L. A. Mocellin M. Acosta P. Caballero M. T. Libster R. (2016). IL-8/IL-17 gene variations and the susceptibility to severe viral bronchiolitis. *Epidemiol. Infect.* 145 642–646. 10.1017/S0950268816002648 27890033 PMC9507717

[B64] Pope C. A. Dockery D. W. (2006). Health effects of fine particulate air pollution: lines that connect. *J. Air Waste Manag. Assoc.* 56 709–742. 10.1080/10473289.2006.10464485 16805397

[B65] Quintana-belmares R. Vega E. Vázquez-lópez I. Rojas-bracho L. López-villegas M. T. Vadillo-ortega F. (2015). TNFα and IL-6 responses to particulate matter in vitro: variation according to PM Size, season, and polycyclic aromatic hydrocarbon and soil content. *Environ. Health Perspect.* 124 406–412. 10.1289/ehp.1409287 26372663 PMC4829995

[B66] Ray A. Raundhal M. Oriss T. B. Ray P. Wenzel S. E. Wenzel S. (2016). Current concepts of severe asthma. *J. Clin. Invest.* 126 2394–2403. 10.1172/JCI84144 27367183 PMC4922699

[B67] Robinson C. L. Baumann L. M. Gilman R. H. Romero K. Combe J. M. Cabrera L. (2012). The Peru Urban versus Rural Asthma (PURA) Study: methods and baseline quality control data from a cross-sectional investigation into the prevalence, severity, genetics, immunology and environmental factors affecting asthma in adolescence in Peru. *BMJ Open* 2:e000421 10.1136/bmjopen-2011-000421 PMC3289983 22357570

[B68] Schins R. P. van Hartingsveldt B. Borm P. J. (1996). Ex vivo cytokine release from whole blood. *Exp. Toxicol. Pathol.* 48 494–496. 10.1016/S0940-2993(96)80064-9 8954330

[B69] Shuster-Meiseles T. Shafer M. M. Heo J. Pardo M. Antkiewicz D. S. Schauer J. J. (2016). ROS-generating/ARE-activating capacity of metals in roadway particulate matter deposited in urban environment. *Environ. Res.* 146 252–262. 10.1016/j.envres.2016.01.009 26775006

[B70] Silbajoris R. Osornio-Vargas A. R. Simmons S. O. Reed W. Bromberg P. A. Dailey L. A. (2011). Ambient particulate matter induces interleukin-8 expression through an alternative NF-κB (nuclear factor-kappa B) mechanism in human airway epithelial cells. *Environ. Health Perspect.* 119 1379–1383. 10.1289/ehp.1103594 21665565 PMC3230452

[B71] Simpson J. L. Brooks C. Douwes J. (2008). Innate immunity in asthma. *Paediatr. Respir. Rev.* 9 263–270. 10.1016/j.prrv.2008.05.007 19026367

[B72] Sioutas C. Delfino R. J. Singh M. (2005). Exposure assessment for atmospheric ultrafine particles (UFPs) and implications in epidemiologic research. *Environ. Health Perspect.* 113 947–955. 10.1289/ehp.7939 16079062 PMC1280332

[B73] Steerenberg P. A. Zonnenberg J. A. Dormans J. A. Joon P. N. Wouters I. M. van Bree L. (1998). Diesel exhaust particles induced release of interleukin 6 and 8 by (primed) human bronchial epithelial cells (BEAS 2B) in vitro. *Exp. Lung Res.* 24 85–100. 10.3109/01902149809046056 9457471

[B74] Stenfors N. Nordenhäll C. Salvi S. S. Mudway I. Söderberg M. Blomberg A. (2004). Different airway inflammatory responses in asthmatic and healthy humans exposed to diesel. *Eur. Respir. J.* 23 82–86. 10.1183/09031936.03.00004603 14738236

[B75] Takeuchi O. Akira S. (2010). Pattern recognition receptors and inflammation. *Cell* 140 805–820. 10.1016/j.cell.2010.01.022 20303872

[B76] Tang R. B. Chen S. J. (2000). Evaluation of serum interleukin-8 as a marker of disease activity in acute asthma in children. *J. Asthma* 37 409–413. 10.3109/02770900009055466 10983618

[B77] Tétreault L. -F. Doucet M. Gamache P. Fournier M. Brand A. Kosatsky T. (2016). Severe and moderate asthma exacerbations in asthmatic children and exposure to ambient air pollutants. *Int. J. Environ. Res. Public Health* 13:771 10.3390/ijerph13080771 PMC4997457 27490556

[B78] Turkeli A. Yilmaz O. Taneli F. Horasan G. D. Kanik E. T. Kizilkaya M. (2015). IL-5, IL-8 and MMP -9 levels in exhaled breath condensate of atopic and nonatopic asthmatic children. *Respir. Med.* 109 680–688. 10.1016/j.rmed.2015.04.004 25937050

[B79] Ueno T. Kataoka M. Hirano A. Iio K. Tanimoto Y. Kanehiro A. (2008). Inflammatory markers in exhaled breath condensate from patients with asthma. *Respirology* 13 654–663. 10.1111/j.1440-1843.2008.01315.x 18513240

[B80] Underhill L. Bose S. Williams D. Romero K. Malpartida G. Breysse P. (2015). Association of roadway proximity with indoor air pollution in a peri-urban community in lima, peru. *Int. J. Environ. Res. Public Health* 12 13466–13481. 10.3390/ijerph121013466 26516875 PMC4627043

[B81] Venn A. J. Lewis S. A. Cooper M. Hubbard R. Britton J. (2001). Living near a main road and the risk of wheezing illness in children. *Am. J. Respir. Crit. Care Med.* 164 2177–2180. 10.1164/ajrccm.164.12.2106126 11751183

[B82] Veranth J. M. Moss T. A. Chow J. C. Labban R. Nichols W. K. Walton J. C. (2006). Correlation of in vitro cytokine responses with the chemical composition of soil-derived particulate matter. *Environ. Health Perspect.* 114 341–349. 10.1289/ehp.8360 16507455 PMC1392226

[B83] Williams L. K. Ownby D. R. Maliarik M. J. Johnson C. C. (2005). The role of endotoxin and its receptors in allergic disease. *Ann. Allergy Asthma Immunol.* 94 323–332. 10.1016/S1081-1206(10)60983-0 15801242 PMC1351105

[B84] Wu D. Tan W. Zhang Q. Zhang X. Song H. (2013). Effects of ozone exposure mediated by BEAS-2B cells on T cells activation: a possible link between environment and asthma. *Asian Pac. J. Allergy Immunol.* 32 25–33. 10.12932/AP0316.32.1.2014 24641287

[B85] Wu W. Muller R. Berhane K. Fruin S. Liu F. Jaspers I. (2014). Inflammatory response of monocytes to ambient particles varies by highway proximity. *Am. J. Respir. Cell Mol. Biol.* 10.1165/rcmb.2013-0265OC PMC4291543 24895888

[B86] Zhang J. J. McCreanor J. E. Cullinan P. Chung K. F. Ohman-Strickland P. Han I.-K. (2009). Health effects of real-world exposure to diesel exhaust in persons with asthma. *Res. Rep. Health. Eff. Inst.* 138 5–109; discussion 111–123. 19449765

[B87] Zhu Y. Hinds W. C. Kim S. Sioutas C. (2002). Concentration and size distribution of ultrafine particles near a major highway. *J. Air Waste Manag. Assoc.* 52 1032–1042. 10.1080/10473289.2002.10470842 12269664

